# Clinical Profile of AXS‐05 in Treating Alzheimer’s Disease Agitation: Results from the Phase 2/3 Development Program

**DOI:** 10.1002/alz.086940

**Published:** 2025-01-09

**Authors:** Jeffrey L. Cummings, George T Grossberg, Caroline Streicher, Herriot Tabuteau

**Affiliations:** ^1^ University of Nevada, Las Vegas, NV USA; ^2^ Saint Louis University Hospital, St. Louis, MO USA; ^3^ Axsome Therapeutics, New York, NY USA

## Abstract

**Background:**

Patients with Alzheimer’s disease (AD) often experience burdensome neuropsychiatric symptoms, including agitation which occurs in both home and long‐term care (LTC) facilities, and is associated with substantial increases in caregiver burden and LTC placements. AXS‐05 (45‐mg dextromethorphan/105‐mg bupropion), a novel, oral NMDA receptor antagonist and sigma‐1 receptor agonist, approved by the FDA for major depressive disorder, is being investigated for treatment of AD agitation (ADA). AXS‐05 has been evaluated in 2 randomized, double‐blind studies: Phase 2 ADVANCE‐1 (NCT03226522); Phase 3 ACCORD (NCT04797715).

**Method:**

ADVANCE‐1/ACCORD enrolled patients (65‐90 y) with a diagnosis of probable AD using 2011 NIA‐AA diagnostic criteria. ADVANCE‐1 evaluated the acute effects of AXS‐05 on improving ADA. Patients were randomized 1:1:1 for 5 weeks (AXS‐05 or bupropion [105‐mg] or matching placebo). Primary endpoint: change from baseline in the Cohen‐Mansfield Agitation Inventory (CMAI) total score. ACCORD evaluated the effects of AXS‐05 in preventing ADA relapse using a randomized discontinuation design; it comprised an open‐label period (OLP; ≤9 weeks) until response was sustained (≥4 consecutive weeks). In the double‐blind period (DBP), patients were randomized 1:1 (AXS‐05/placebo) for up to 26 weeks in the DBP or until relapse. Primary endpoint: time‐to‐relapse.

**Result:**

ADVANCE‐1 comprised 357 patients in the modified intent‐to‐treat population: AXS‐05 (n = 152) bupropion (n = 49), placebo (n = 156) (mean baseline CMAI total scores = 60.7, 66.1, 59.4, respectively). At Week 5, AXS‐05 significantly reduced CMAI total score by 15.4 points vs bupropion (10.0 points; P<.001) and placebo (11.5 points; P = .010). In ADVANCE‐1, common adverse events (AEs; ≥3%) included somnolence (8.2%), dizziness (6.3%), and diarrhea (4.4%). ACCORD comprised 178 patients in the OLP (mean CMAI = 70.9) and 108 patients in the DBP (AXS‐05, n = 53; placebo, n = 55) (mean CMAI = 43.7). AXS‐05 increased time‐to‐relapse vs placebo (hazard ratio = 0.275; P = .014). AXS‐05 was associated with lower relapse rates vs placebo (7.5% vs 25.9%; P<.05). In ACCORD, common AEs (≥3%) included dizziness (9.6%), diarrhea (4.5%), and fall (5.1%). AXS‐05 was not associated with cognitive impairment or sedation in either study.

**Conclusion:**

AXS‐05 demonstrated a substantial, rapid reduction in ADA vs controls after 5 weeks of treatment, increased time‐to‐relapse vs placebo, and was well‐tolerated, supporting its development as a promising treatment option for ADA.

